# Postoperative pain trajectories and associated factors in patients undergoing lung cancer surgery: a scoping review

**DOI:** 10.3389/fpsyg.2026.1906338

**Published:** 2026-09-09

**Authors:** Jinchun Liu, Yu Zhu, Fan Wang, Luan Xiang, Xueqin Wang

**Affiliations:** 1 Department of Cardiothoracic Surgery, General Hospital of Central Theater Command, Wuhan, Hubei, China; 2 School of Medicine, Wuhan University of Science and Technology, Wuhan, Hubei, China

**Keywords:** lung cancer, pain, pain trajectories, scoping review, associated factors

## Abstract

**Objectives:**

This scoping review aimed to map the available evidence on postoperative pain trajectories and associated factors in patients undergoing lung cancer surgery and to inform precision pain management in clinical practice.

**Methods:**

PubMed, Web of Science, the Cochrane Library, Embase, CINAHL, CNKI, Wanfang, VIP, and the China Biology Medicine database (CBM) were searched from inception to January 31, 2026. Eligible studies were screened, charted, and narratively synthesized.

**Results:**

Eleven studies were included, reporting 35 study-specific trajectory classes, with two to four classes identified per study. These trajectories could be broadly grouped into trajectories based on pain intensity and recovery outcomes, trajectories based on pain threshold changes, and trajectories characterized by pain context or special patterns of evolution. Fourteen assessment tools were identified and classified into five categories: pain measurement instruments, generic pain scales, pain-specific scales, psychological and emotional scales, and sleep-related scales. Associated factors clustered into four domains: physiological, surgery-related, analgesic, and cognitive-psychological factors.

**Conclusion:**

Postoperative pain trajectories following lung cancer surgery are heterogeneous and study-specific. Future studies should standardize trajectory measurement and reporting, externally validate risk-stratification approaches, and prospectively evaluate the clinical effectiveness of trajectory-informed care.

**Systematic review registration:**

https://archive.org/details/osf-registrations-s6u7q-v1.

## 1 Introduction

Lung cancer remains the most commonly diagnosed cancer worldwide, accounting for 12.4% of all new cancer cases (Bray et al., 2024). Surgical resection is the treatment of choice for patients with early- and intermediate-stage lung cancer (Chinese Society of Oncology, Chinese Medical Association, 2025). However, because of surgical tissue trauma and postoperative inflammatory responses, postoperative pain is highly prevalent after lung cancer surgery. The incidence of moderate-to-severe pain within the first 24 h after surgery has been reported to be 28.3% (Wang et al., 2024), and inadequately controlled acute pain may further evolve into chronic postsurgical pain, with a reported incidence of 35.3% (Chen et al., 2023). Postoperative pain causes considerable suffering, delays recovery, and impairs long-term quality of life (Lei et al., 2023).

Pain has long been regarded as the “fifth vital sign,” yet its management remains challenging in clinical practice because pain evolves dynamically and is inherently subjective. Pain trajectories refer to dynamic and continuous patterns of change in pain intensity, duration, and characteristics over time and have been increasingly used to explain heterogeneity in postoperative pain outcomes (Vasilopoulos et al., 2021). They provide an important basis for individualized analgesic planning, identification of key intervention windows, and risk stratification for adverse pain outcomes. However, the current evidence remains inconsistent with respect to trajectory models, trajectory nomenclature, measurement time points, and associated factors, thereby limiting evidence synthesis and clinical translation.

Against this background, this scoping review was conducted in accordance with the framework proposed by Arksey and O'Malley (2005) and the updated methodological guidance for scoping reviews published by the Joanna Briggs Institute (Peters et al., 2021). The aim was to systematically map the available evidence on postoperative pain trajectories and associated factors in patients undergoing lung cancer surgery, summarize the reported trajectory patterns and assessment tools, and provide evidence to support more precise postoperative pain management in clinical practice. The review protocol was registered in the Open Science Framework (OSF) (DOI: 10.17605/OSF.IO/S6U7Q).

## 2 Methods

### 2.1 Design and reporting guideline

The reporting of this review followed the Preferred Reporting Items for Systematic Reviews and Meta-Analyses extension for Scoping Reviews (PRISMA-ScR) (Tricco et al., 2018). A formal study-level methodological appraisal using tools such as the JBI critical appraisal checklists was considered during the review design. However, given that the primary purpose of this scoping review was to map the breadth, characteristics, and methodological heterogeneity of the available evidence rather than to estimate pooled effects or make judgments about certainty of evidence, formal critical appraisal was not undertaken. Instead, methodological characteristics relevant to interpretation, including study design, sample size, setting, follow-up duration, and trajectory-modeling approach, were systematically charted and considered in the narrative synthesis and interpretation of the findings.

### 2.2 Review questions

The review sought to address the following questions: (1) What analytical approaches and trajectory categories have been used to characterize postoperative pain trajectories in patients undergoing lung cancer surgery? (2) What tools have been used to assess postoperative pain and related symptoms in this population? (3) What factors have been reported to be associated with postoperative pain trajectories after lung cancer surgery?

### 2.3 Eligibility criteria

Eligibility criteria were defined according to the participants–concept–context (PCC) framework (Peters et al., 2021). Studies were eligible if they: (1) included adult patients with lung cancer who underwent surgical treatment; (2) focused on postoperative pain trajectories after lung cancer surgery; and (3) reported information on trajectory categories, assessment tools, or associated factors. Conference abstracts, reviews, news reports, non-Chinese and non-English articles, duplicate publications, and studies without accessible full text were excluded.

### 2.4 Search strategy

PubMed, Web of Science, the Cochrane Library, Embase, CINAHL, CNKI, Wanfang, VIP, and the China Biology Medicine database (CBM) were searched from inception to January 31, 2026. For the English databases, a combination of subject headings and free-text terms was used. Using PubMed as an example, the search strategy covered four main concepts: lung cancer, surgery, postoperative pain, and pain trajectories or longitudinal trajectory modeling. For Chinese databases, corresponding Chinese search terms relating to lung cancer, thoracic surgery, postoperative pain, and trajectory analysis were used. Manual backward citation searching was also conducted to identify additional relevant studies.

### 2.5 Study selection and data charting

All retrieved records were imported into EndNote 20 for deduplication. Two reviewers independently screened titles and abstracts against the eligibility criteria, followed by full-text review of potentially relevant studies. Disagreements were resolved through discussion with a third reviewer. Data extraction was performed independently by two reviewers using a standardized charting form. Extracted information included author, publication year, country, study design, sample size, trajectory model, follow-up schedule, assessment tools, number of trajectories, trajectory groups, and associated factors.

## 3 Results

### 3.1 Study selection

**Figure 1:**
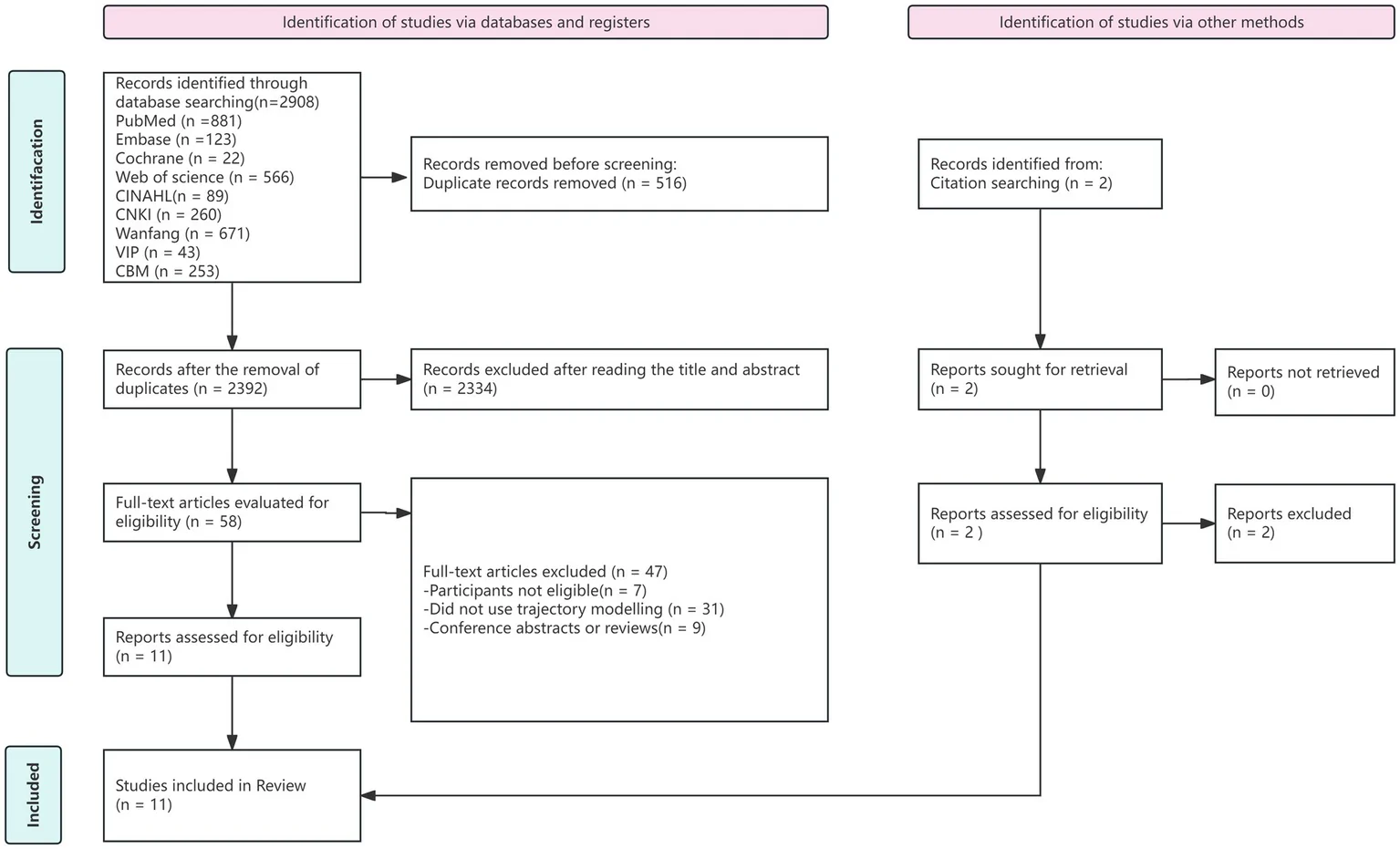
Flow diagram of study selection.

A total of 2,908 records were identified. After removal of duplicates, 2,392 records remained for title and abstract screening. Of these, 2,334 records were excluded as clearly irrelevant. Fifty-eight full-text articles were assessed for eligibility, and 47 were excluded because of ineligible populations, inappropriate methods, or unsuitable publication formats. Eleven studies were ultimately included in the review (see Figure 1).

### 3.2 Characteristics of included studies

The 11 included studies were published between 2020 and 2026. Six studies were conducted in China, and one each in Japan, Denmark, France, Canada, and Norway. All included studies used a prospective cohort design. The basic characteristics of the included studies are presented in Table 1.

**Table 1:** Characteristics of the included studies (*n* = 11).

Author/year	Country	Study design	Sample size	Trajectory model	Follow-up	Assessment instruments	No. of trajectories	Trajectory groups (proportion)	Associated factors
Zhu et al. (2025)	China	Prospective cohort	325	GBTM	On admission; postoperative day 1; postoperative day 3	1) Handheld pain-threshold meter 2) GAD-7 3) SRSS 4) PCS	4	1) High pain threshold smooth decline group (10.8%) 2) Medium-high pain threshold elastic decline group (26.5%) 3) Medium pain threshold smooth decline group (47.4%) 4) Low pain threshold smooth decline group (15.4%)	Gender, alcohol consumption, diabetes comorbidity, and psychological scales (GAD-7, SRSS, PCS)
Liu et al. (2026)	China	Prospective cohort	439	GMM	Preoperatively to 30 days after discharge	1) NRS	2	1) Non-recovery/persistent moderate pain (14.8%) 2) Recovery/mild pain decline (85.2%)	Subcutaneous fat index, preoperative pain severity, length of hospital stay
Xie et al. (2025)	China	Prospective cohort	220	GBTM	Postoperative days 1–5	1) NRS 2) HADS 3) PCS 4) PBPI	3	1) Mild pain (49.1%) 2) Mild-to-moderate pain (47.3%) 3) Moderate-to-severe pain (3.6%)	Operative duration, chest drain retention time, anxiety, depression, pain catastrophizing, pain beliefs and perceptions
Wang et al. (2025)	China	Prospective cohort	178	LPA	Postoperative days 1–7, 3 times daily	1) NRS 2) HADS 3) PCS	3	1) Mild to complete relief (52.4%) 2) Moderate-to-mild pain (31.9%) 3) High-to-moderate pain (15.7%)	For the moderate-to-mild pain group: sex, morphine dose, drain retention duration, HADS score, PCS score. For the high-to-moderate pain group: sex, operative duration, drain retention duration, HADS score, PCS score.
He et al. (2025)	China	Prospective cohort	321	LCMM	Postoperative days 1–6	1) NRS 2) APAIS	4	1) Persistent mild pain (52.0%) 2) Mild-to-moderate pain with relief (17.1%) 3) Pain aggravation followed by relief (15.0%) 4) Moderate pain with relief (15.9%)	Sex, surgical approach, intercostal nerve block, use of analgesic pump, preoperative anxiety, number of incisions
Zhao et al. (2024)	China	Prospective cohort	231	GMM	Postoperative days 1, 2, 3, 30, 60 and 90	1) NRS 2) HADS 3) PCS	4	1) Low-level rest pain (89.18%) 2) High-level rest pain (10.82%) 3) Low-level movement-evoked pain (82.25%) 4) High-level movement-evoked pain (17.75%)	High-level rest pain: route of analgesia and chest tube diameter. High-level movement-evoked pain: sex and chest tube diameter.
Saito et al. (2023)	Japan	Prospective cohort	75	JRM	Once preoperatively; daily during week 1; every 2 days during week 2; weekly during week 3; daily after discharge until first outpatient follow-up	1) NRS	2	1) Rebound pain (53.3%) 2) Non-rebound pain (46.7%)	Pain severity on postoperative day 4
Danielsen et al. (2024)	Denmark	Prospective cohort	85	GBTM	1–3 days preoperatively; 6 and 12 months after surgery	1) Surgical-site pain sensitivity tester 2) NRS 3) NPSI 4) HADS	3	1) Complete recovery (50%) 2) protracted recovery (37%) 3) Incomplete recovery (13%)	Acute postoperative pain intensity, age, neuropathic pain symptoms
Dorges et al. (2022)	France	Prospective cohort	181	HCA	Postoperative days 1–5	1) NRS 2) DN4	4	1) Significant pain (29.8%) 2) Low pain (32.6%) 3) Rapid pain decline (22.7%) 4) Rapid pain increase (14.9%)	Preoperative pain, wound length, ASA class
Liu et al. (2021)	Canada	Prospective cohort	237	GMM	Before surgery; postoperative days 1–3; 3, 6 and 12 months after surgery	1) NRS 2) HADS 3) PSQI 4) PASS-20 5) PCS	3	1) Mild pain intensity across the 12 month period (78.1%) 2) Moderate pain intensity immediately after surgery which decreases markedly by 3 months and remains low at the 12 month follow-up (7.5%) 3) Moderate pain intensity immediately after surgery which persists at 12 months. (8.4%)	Immediate postoperative pain intensity, preoperative pain catastrophizing
Gjeilo et al. (2020)	Norway	Prospective cohort	264	LCMM	Before surgery; 1, 5, 9 and 12 months after surgery	1) BPI 2) NRS	3	1) No pain (8%) 2) Pain increasing then declining (mean pain: 64%; worst pain: 74%) 3) Persistently high pain (mean pain: 28%; worst pain: 18%)	Comorbidity score, preoperative analgesic use, preoperative psychotropic medication use, surgical type

GBTM, group-based trajectory modeling; GMM, growth mixture modeling; LPA, latent profile analysis; LCMM, latent class mixed modeling; JRM, joinpoint regression modeling; HCA, hierarchical cluster analysis; POD, postoperative day; NRS, Numerical Rating Scale; HADS, Hospital Anxiety and Depression Scale; PCS, Pain Catastrophizing Scale; PBPI, Pain Beliefs and Perceptions Inventory; APAIS, Amsterdam Preoperative Anxiety and Information Scale; NPSI, Neuropathic Pain Symptom Inventory; DN4, Douleur Neuropathique 4 questionnaire; PASS-20, Pain Anxiety Symptoms Scale-20; PSQI, Pittsburgh Sleep Quality Index; BPI, Brief Pain Inventory.

### 3.3 Number and categories of pain trajectories

Trajectory analyses were primarily based on longitudinal data-driven modeling aimed at identifying latent subgroups of pain evolution. Reported approaches included growth mixture modeling (GMM) (Liu et al., 2026; Zhao et al., 2024; Liu et al., 2021), group-based trajectory modeling (GBTM) (Zhu et al., 2025; Xie et al., 2025; Danielsen et al., 2024), latent profile analysis (LPA) (Wang et al., 2025), latent class mixed modeling (LCMM) (He et al., 2025; Gjeilo et al., 2020), joinpoint regression modeling (JRM) (Saito et al., 2023), and hierarchical cluster analysis (HCA) (Dorges et al., 2022). Across the included studies, 35 study-specific trajectory classes were reported, with each study identifying two to four classes.

These trajectories could be grouped into three broad categories. First, trajectories based on pain intensity and recovery outcomes included classes such as persistent moderate pain without recovery, mild pain with recovery, persistent mild pain, persistent moderate-to-severe pain, and incomplete recovery (Liu et al., 2026; Xie et al., 2025; Wang et al., 2025; He et al., 2025; Danielsen et al., 2024; Dorges et al., 2022; Liu et al., 2021; Gjeilo et al., 2020). Second, trajectories based on changes in pain thresholds were reported in one study and reflected different patterns of perioperative pain sensitivity (Zhu et al., 2025). Third, trajectories based on pain context or special patterns of evolution included rest pain versus movement-evoked pain trajectories and rebound versus non-rebound pain trajectories (Zhao et al., 2024; Saito et al., 2023).

### 3.4 Pain and symptom assessment tools

A total of 14 pain and symptom assessment tools were identified and could be classified into five categories. Pain measurement instruments included a handheld pressure pain threshold device and an instrument for assessing surgical-site pain sensitivity (Zhu et al., 2025; Danielsen et al., 2024). Generic pain scales included the Numerical Rating Scale (NRS) (Liu et al., 2026; Xie et al., 2025; Wang et al., 2025; He et al., 2025; Zhao et al., 2024; Saito et al., 2023; Danielsen et al., 2024; Dorges et al., 2022; Liu et al., 2021; Gjeilo et al., 2020), and the Brief Pain Inventory (BPI) (Gjeilo et al., 2020). Pain-specific scales included the Pain Catastrophizing Scale (PCS), Pain Beliefs and Perceptions Inventory (PBPI), Neuropathic Pain Symptom Inventory (NPSI), and DN4 questionnaire (Xie et al., 2025; Wang et al., 2025; Zhao et al., 2024; Danielsen et al., 2024; Dorges et al., 2022; Liu et al., 2021). Psychological and emotional scales included the Generalized Anxiety Disorder-7 (GAD-7), the Amsterdam Preoperative Anxiety and Information Scale (APAIS), the Hospital Anxiety and Depression Scale (HADS), and the Pain Anxiety Symptoms Scale-20 (PASS-20) (Zhu et al., 2025; He et al., 2025; Danielsen et al., 2024; Liu et al., 2021). Sleep-related scales included the Self-Rating Sleep Scale (SRSS) and the Pittsburgh Sleep Quality Index (PSQI) (Zhu et al., 2025; Liu et al., 2021).

### 3.5 Factors associated with pain trajectories

A total of 26 associated factors were identified and grouped into four domains. Physiological factors included sex, age, drinking history, comorbidities, and subcutaneous fat index (Zhu et al., 2025; Wang et al., 2025; Zhao et al., 2024; Danielsen et al., 2024; Liu et al., 2026). Surgery-related factors included operative duration, surgical approach, duration of chest drainage, chest tube diameter, number of incisions, incision length, length of stay, and ASA classification (Xie et al., 2025; Wang et al., 2025; He et al., 2025; Zhao et al., 2024; Dorges et al., 2022; Gjeilo et al., 2020). Analgesic factors included morphine dose, intercostal nerve block, use of analgesia pumps, analgesic route, preoperative pain, use of analgesics before surgery, preoperative psychotropic medication, early postoperative pain intensity, and neuropathic pain symptoms (Wang et al., 2025; He et al., 2025; Zhao et al., 2024; Dorges et al., 2022; Gjeilo et al., 2020; Danielsen et al., 2024; Liu et al., 2021; Saito et al., 2023). Cognitive-psychological factors included pain catastrophizing, pain beliefs and perceptions, sleep, anxiety and depression (Zhu et al., 2025; Xie et al., 2025; Wang et al., 2025; He et al., 2025; Liu et al., 2021).

## 4 Discussion

### 4.1 Identifying pain trajectories may facilitate precision pain management

Postoperative pain after lung cancer surgery does not follow a single linear course but instead comprises multiple dynamic patterns of change. The trajectory solutions identified across studies were derived using different statistical approaches with substantially different theoretical foundations and assumptions and therefore should not be interpreted as a unified or directly comparable taxonomy of postoperative pain phenotypes. Moreover, the reporting of model-fit and classification-quality indices—including the Bayesian information criterion, entropy, posterior classification probabilities, and class-specific sample sizes—was inconsistent across studies, further limiting the robustness, cross-study comparability, and clinical interpretability of the trajectory findings. Accordingly, these trajectory classes are best regarded as study-specific solutions generated under particular sample and methodological conditions.

Beyond differences in statistical modeling, variation in study design and perioperative clinical context also contributed to trajectory heterogeneity. Follow-up periods ranged from several postoperative days to 12 months. Short-term studies primarily captured the resolution, fluctuation, or rebound of acute pain, whereas longer-term studies were more likely to reflect persistent pain and the transition from acute to chronic postsurgical pain. Pain outcomes also varied across studies and included pain intensity, pain thresholds, pain at rest, and movement-evoked pain. Differences in assessment timing and frequency may likewise have influenced the shape and number of identified trajectories. Furthermore, variation in surgical approach, regional anesthesia, route of analgesic administration, opioid exposure, multimodal analgesia, chest drain management, and implementation of Enhanced Recovery After Surgery pathways may have affected postoperative pain severity and recovery patterns.

Despite this heterogeneity, the reported trajectories could still be broadly grouped into three categories: trajectories characterized by pain intensity and recovery outcomes, trajectories reflecting dynamic changes in pain thresholds, and trajectories defined by pain context or distinctive patterns of evolution. The value of this higher-order classification lies in identifying recurrent patterns of pain evolution, high-risk subgroups, and potential windows for intervention.

### 4.2 Multidimensional assessment tools may improve the precision and clinical applicability of pain evaluation

Postoperative pain after lung cancer surgery is a multidimensional experience involving both somatic sensations and psychological-cognitive components. Accurate assessment is therefore a prerequisite for effective intervention and requires multidimensional tools to support both quantification and phenotypic characterization. In the present review, five categories of pain-related assessment tools were identified.

Among generic pain scales, the NRS remains the most commonly used instrument because of its simplicity, efficiency, and high patient acceptability. However, it relies heavily on self-report and may be less precise than instrument-based measures when characterizing certain aspects of pain sensitivity. Instrument-based pressure pain threshold testing can provide objective information on pain sensitivity, but its routine clinical use remains limited because of equipment requirements and feasibility constraints.

Pain-specific instruments may offer a more comprehensive assessment of sensory, affective, and functional dimensions of pain and can therefore support finer phenotyping of pain subtypes, although their larger number of items may increase respondent burden. Moreover, pain is closely intertwined with psychological status and sleep quality, often forming a vicious cycle of pain, psychological distress, and sleep disturbance (Zambelli et al., 2021). Accordingly, assessment frameworks for postoperative pain should ideally incorporate psychological and sleep-related dimensions. In clinical practice, tool selection should be guided by the patient’s condition, cognitive capacity, and the clinical purpose of assessment. Concise and repeatable tools are more practical for high-frequency perioperative monitoring, whereas patients at risk of pain persistence or chronicity may require additional assessment of psychological, sleep-related, and cognitive dimensions.

### 4.3 Exploring factors associated with pain trajectories may improve risk prediction and targeted management

The present review indicates that postoperative pain trajectories following lung cancer surgery are associated with four major domains: physiological characteristics, surgical and perioperative factors, analgesic strategies, and cognitive–psychological factors. However, the consistency of reporting across studies varies substantially across these domains.

Female sex, greater early postoperative pain intensity, pain catastrophizing, and psychological distress, including anxiety and depressive symptoms, were repeatedly identified across multiple studies and may therefore represent relatively consistent indicators of unfavorable postoperative pain trajectories. Previous studies have demonstrated that women tend to report higher pain intensity and greater sensitivity to painful stimuli across various acute and chronic pain conditions (Xiao et al., 2023). Similarly, greater pain severity during the early postoperative period has been associated with delayed pain resolution, pain rebound, and persistent postsurgical pain. A systematic review including 56 studies further confirmed that preoperative pain and higher acute postoperative pain intensity were associated with an increased risk of chronic postsurgical pain (Clephas et al., 2023). Pain catastrophizing may enhance pain-related threat appraisal and amplify the subjective experience of pain severity (Lersch et al., 2023). Meanwhile, anxiety and depressive symptoms may increase pain vigilance, exacerbate emotional distress, and interfere with adaptive recovery behaviors. These psychological processes are consistent with the fear-avoidance model of pain (Vlaeyen and Linton, 2000), which suggests that negative interpretations of pain and catastrophic beliefs may lead individuals to perceive pain as a threatening experience, resulting in fear of movement and avoidance behaviors. Persistent avoidance may subsequently contribute to physical deconditioning, psychological distress, and pain persistence, thereby creating a potential vicious cycle of chronic pain and functional impairment. However, this psychological–behavioral pathway has not yet been directly investigated within postoperative pain trajectory studies among patients undergoing lung cancer surgery. Therefore, the combination of female sex, high early postoperative pain intensity, and adverse psychological status may characterize a subgroup with increased vulnerability to unfavorable pain trajectories, although this profile should not yet be considered a validated predictive model.

In contrast, most surgical and physiological factors were reported in only one or a limited number of studies and should therefore be interpreted as clinically relevant factors with preliminary evidence rather than established predictors. Nevertheless, from a thoracic surgical perspective, these factors may represent important opportunities for perioperative optimization because several are potentially modifiable. Differences between minimally invasive and open surgical approaches, including the extent of tissue trauma, incision characteristics, and intercostal nerve manipulation, may contribute to heterogeneous postoperative pain patterns. Although VATS avoids the rib spreading associated with conventional thoracotomy, repeated movement and pressure of thoracoscopic instruments through intercostal ports may still cause intercostal nerve injury (Miyazaki et al., 2011; Miyazaki et al., 2024). Port characteristics, intercostal nerve injury, and chest drain placement and duration may further influence pain at rest, movement-evoked pain, and early functional recovery; prospective evidence indicates that chest tube insertion itself may impair intercostal nerve function and contribute to postoperative pain (Miyazaki et al., 2014). Perioperative strategies, including regional anesthesia, opioid-sparing multimodal analgesia, early chest drain removal, and early mobilization, are recommended in procedure-specific VATS analgesia and ERAS/ESTS lung surgery guidelines and may modify early postoperative pain and recovery patterns (Feray et al., 2022; Batchelor et al., 2019). Importantly, perioperative factors may interact with psychological vulnerability rather than operate independently. For example, patients with higher levels of pain catastrophizing or anxiety may interpret chest drain-related discomfort or movement-evoked pain as threatening signals, leading to reduced participation in coughing exercises, mobilization, and pulmonary rehabilitation (Grosen et al., 2014).

Other variables, including alcohol consumption, diabetes, subcutaneous fat index, preoperative psychotropic medication use, sleep status, and pain beliefs and perceptions, were associated with pain trajectories in individual studies but have not yet demonstrated consistent replication across cohorts. These variables should therefore be regarded as exploratory correlates rather than independent indicators for routine clinical risk stratification.

### 4.4 Clinical implications for trajectory-informed perioperative pain management

Based on the factors identified above, pain trajectory assessment could be translated into a staged and operational perioperative pain management pathway. During the preoperative phase, preliminary risk stratification could incorporate female sex, a history of chronic preoperative pain, pain catastrophizing, anxiety or depressive symptoms, and the anticipated extent of surgical trauma. These factors could then be used to determine the intensity and focus of postoperative pain monitoring.

Postoperative days 1–3 may represent a key surveillance window, during which analgesic regimens can be optimized and patients at risk of unfavorable pain trajectories can be identified early. If pain remains inadequately controlled during this period and is not followed by intensified assessment and timely adjustment of analgesic management, the risk of persistent or chronic postsurgical pain may increase. Postoperative days 3–4 may also represent an important period for pain rebound, suggesting that clinicians should assess the need to modify multimodal analgesic regimens before the effect of a single analgesic intervention diminishes or the intervention is discontinued (Saito et al., 2023).

From the perspective of pain context, the trajectories of pain at rest and movement-evoked pain identified by Zhao et al. (2024) have particularly direct clinical relevance. Pain at rest mainly reflects ongoing nociceptive stimulation from the surgical incision and chest drain, as well as the adequacy of baseline analgesic coverage. In contrast, movement-evoked pain commonly occurs during turning, coughing, ambulation, and pulmonary rehabilitation and is closely related to lung re-expansion, effective sputum clearance, tolerance of early mobilization, and functional recovery. In patients undergoing thoracic surgery, poorly controlled movement-evoked pain not only increases subjective distress but may also restrict effective coughing and lung expansion, thereby increasing the risk of atelectasis, impaired secretion clearance, and delayed recovery.

When a patient exhibits features suggestive of an unfavorable pain trajectory, a comprehensive reassessment should be initiated. This reassessment should focus on the adequacy of baseline analgesia, persistent nociceptive stimulation related to chest drainage, waning effects of regional analgesia, and worsening psychological distress. A multidisciplinary team involving thoracic surgeons, anesthesiologists, nurses, and rehabilitation professionals could then develop an individualized management plan based on the reassessment findings. Potential strategies may include optimization of multimodal analgesia, adjustment of analgesic routes or regimens, reassessment of chest drain management and timing of removal, provision of targeted psychological support, and modification of breathing exercises and early mobilization according to the severity of movement-evoked pain.

This pathway could be embedded within Enhanced Recovery After Surgery (ERAS) programs and extended to post-discharge electronic follow-up, thereby facilitating the early identification and timely referral of patients with persistent pain, delayed recovery, or suspected chronic postsurgical pain. However, the current evidence is derived predominantly from small, single-center observational cohorts, and no interventional study has yet demonstrated that such a pathway can alter pain trajectories or improve long-term outcomes. Its clinical effectiveness therefore requires validation in multicenter prospective studies.

## 5 Limitations

Several limitations of this review need to be acknowledged. First, this scoping review was designed to map the overall landscape of existing evidence rather than assess the efficacy of clinical interventions, so formal risk-of-bias appraisal of included studies was not performed. Nevertheless, most included studies were small, single-center prospective cohorts, which may limit the robustness, reproducibility, and generalizability of the reported associations. Second, substantial discrepancies existed across primary studies in statistical modeling schemes, outcome definitions, pain assessment timelines and model selection criteria, making direct cross-comparison of pain trajectory subtypes infeasible. Third, only articles published in Chinese and English were retrieved, which may omit relevant evidence reported in other languages and lead to language bias. Meanwhile, six out of the eleven included studies were conducted in China, meaning the risk predictors summarized herein should be generalized cautiously to medical systems and perioperative management models in other regions.

## 6 Conclusion

This scoping review systematically mapped the available evidence on postoperative pain trajectories in patients undergoing lung cancer surgery and identified multiple heterogeneous and study-specific patterns of pain evolution. The available evidence suggests that incorporating a longitudinal trajectory-based perspective alongside single time-point pain assessment may help identify clinically relevant changes in pain, unfavorable recovery patterns, and patients requiring intensified monitoring, thereby informing the development of individualized, trajectory-informed pain management. However, substantial heterogeneity in modeling approaches, assessment schedules, outcome definitions, and reporting practices limits direct comparisons across studies and the translation of findings into clinical practice. Current research has focused primarily on trajectory identification and associated factors, whereas prospective interventions based on trajectory stratification and external validation of risk-stratification models remain scarce. Future studies should standardize measurement frameworks, model-selection procedures, and reporting practices, and should conduct multicenter external validation and prospective intervention studies to determine the clinical effectiveness of trajectory-informed care and advance precision pain management after lung cancer surgery.

## Data Availability

The original contributions presented in the study are included in the article/Supplementary material, further inquiries can be directed to the corresponding author.
